# Oral Squamous Cell Carcinoma in a Patient with Fanconi Anemia

**DOI:** 10.1155/2021/5571649

**Published:** 2021-06-23

**Authors:** Milla Huuhka, Aaro Turunen

**Affiliations:** ^1^Department of Oral and Maxillofacial Diseases, Turku University Hospital, Turku, Finland; ^2^Department of Oral and Maxillofacial Surgery, Institute of Dentistry, University of Turku, Turku, Finland

## Abstract

Fanconi anemia (FA) is a rare autosomal recessive genetic disorder characterized by different types of malformations, skin lesions, bone marrow failure, and increased risk for both hematological malignancies and solid tumors, especially head and neck squamous cell carcinomas (HNSCC). FA patients may also display a low tolerance to oncologic treatments. The authors present a case of mandibular squamous cell carcinoma in a young FA patient. Because of the aggressive nature of the SCC and complex treatment options, we recommend a strict lifelong follow-up for all FA patients to detect early changes in the oral mucosa.

## 1. Introduction

Fanconi anemia (FA) is a rare genetic disorder with an estimated prevalence of 1 : 160 000 at birth. It is inherited mainly via an autosomal recessive pathway [1]. The disease is characterized by progressive bone marrow failure, hyperpigmentation, pancytopenia, predisposition to both solid tumors and hematological malignancies (i.e., acute myeloid leukemia), and different kinds of congenital malformations [1–3]. Pathogenesis of FA involves biallelic mutations in at least one of the 23 genes responsible for chromosome stability and DNA repair via the FA/BRCA pathway [3].

On most patients, the diagnosis of FA is first suspected in childhood based on hematological abnormalities, growth retardation, and/or congenital malformations. Laboratory analysis by choromosome exposure to cross-linking agents such as diepoxybutane (DEB) or mitomycin C (MMC) reveals complex metaphasic chromatid exchange patterns in the peripheral blood sample, usually leading to FA diagnosis [2]. Because of the progressive nature of FA, the only curative treatment for bone marrow failure is hematopoietic stem cell transplantation (HSCT). After HSCT, patients are susceptible to developing acute or chronic graft versus host disease (GVHD). Chronic GVHD is a known risk factor for malignancies such as OSCC.

FA leads to a 200- to 800-fold increased risk for head and neck squamous cell carcinomas. Majority of the tumors are oral squamous cell carcinomas (OSCC), and the patients are unusually young at disease presentation [3]. As the overall outcome of HSCT has improved during the last decade, the patients with FA live longer lives. Thus, considering the cumulative risk of carcinoma development and increased survival in FA, frequency of OSCCs associated with this rare disorder can be expected to increase in the future [3–5].

## 2. Case Presentation

**Figure 1:**
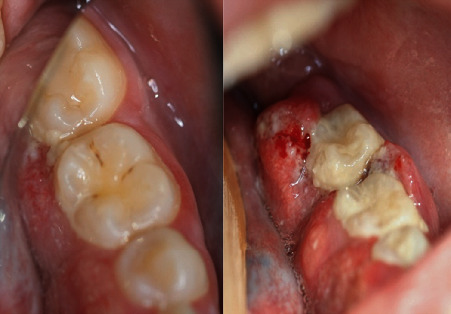
The patient at presentation/biopsy appointment (a) and one month later, before surgery (b). A marked enlargement of the tumor area is shown, with extension to the buccal side of the molar teeth. Plaque accumulation is also detected, owing to pain during brushing. Clinical photography proved difficult due to pain and discomfort affecting the area.

**Figure 2:**
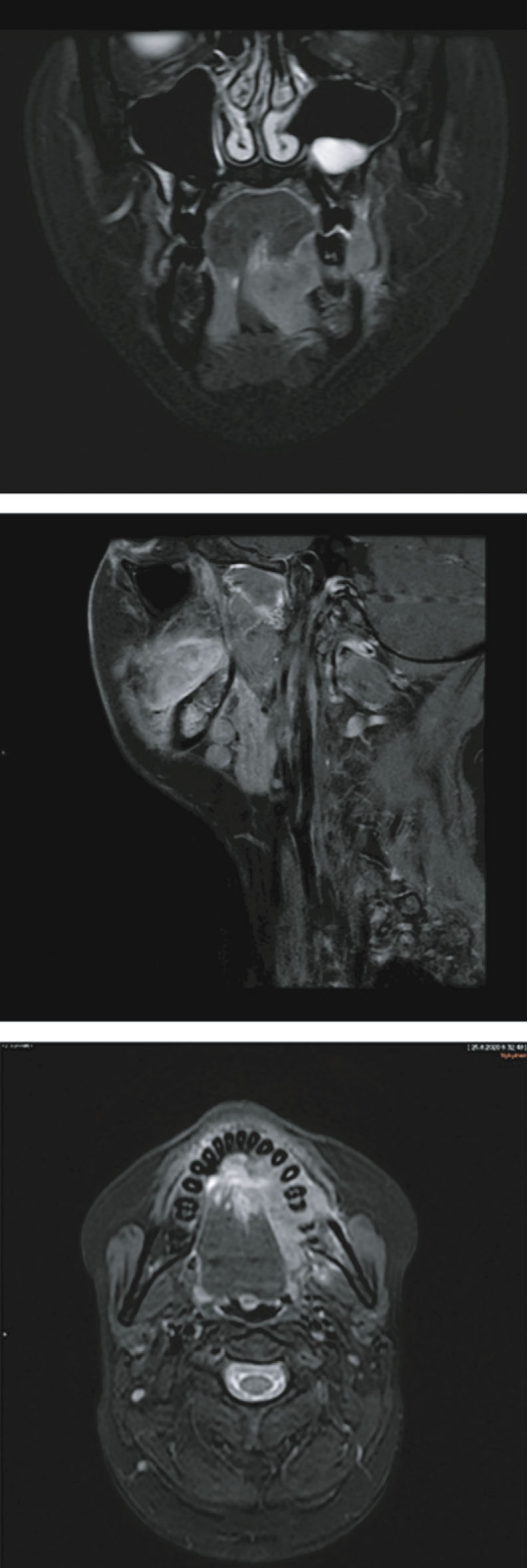
Contrast-enhanced MRI taken after initial examination. The tumor mass is shown infiltrating the mandibular bone, intrinsic musculature of the tongue, and the buccal mucosa, up to the depth of the mylohyoid muscle on the left side. Submandibular pathologic lymph node enlargement is also detected.

**Figure 3:**
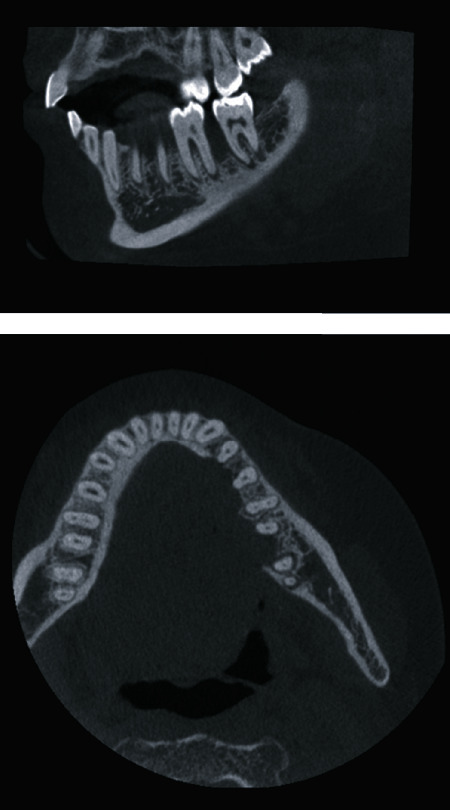
CBCT imaging of the lower jaw. Cortical bone destruction, affecting the lingual cortex that extends from the canine up to the second molar, is shown.

A 28-year-old female with FA was referred to consultation by a local dentist due to an asymptomatic tumor in the lingual side of the patient's left mandibular gingiva (Figure 1). According to the patient, she had noticed the mass 3-4 weeks prior to the visit. The patient did not have a history of smoking, and her alcohol use was minimal. She had undergone a hematopoietic stem cell transplant (HSCT) 14 years prior and had suffered a mild GVHD mainly affecting the skin and lungs. At presentation, her only regular medication was estrogen replacement. Histological examination confirmed squamous cell carcinoma grade 1. A CT scan of the tumor revealed invasion of the mandibular bone and two pathologic lymph nodes in the ipsilateral submandibular space (Figures 2 and 3). The disease was staged T4aN1M0, grade I. HPV testing with p16 and PCR was negative. The patient underwent mandibular resection with fibula osteocutaneous flap reconstruction with concomitant neck dissection, performed at levels Ia-IV. The patient was tracheostomized, and a PEG tube was inserted. A subsequent postoperative chemoradiotherapy to the tumor area to 65 Gy with a simultaneous 63 Gy to the neck metastases and up to 48 Gy to the uninvolved neck area in 2 Gy fractions was administered. Up to two weekly doses of concurrent chemotherapy with 50% reduced dose of cisplatin were administered. However, both doses inflicted major side effects with grade III mucositis, dysphagia, and hair loss, leading to chemotherapy discontinuation. Having completed the radiation treatment regime, the patient is now under follow-up.

## 3. Discussion

Fanconi anemia is a heterogeneous disease, characterized by hematological aberrations and congenital malformations. The patients are divided into different complementation groups in which the phenotype varies from mild to severe. A majority of FA patients develop bone marrow failure and subsequent pancytopenia within their first decade of life. However, an absence of hematological abnormalities, while rare, does not rule out FA [4, 5].

Generally, hematological abnormalities develop within the first decade of life. Currently, the treatment of choice for bone marrow failure is hematopoietic stem cell transplant (HSCT). The prognosis for HSCT has improved over the last decades, leading to increased numbers of patients surviving into adulthood. Thus, more FA patients also develop malignancies such as head and neck squamous cell carcinoma (HNSCC) [6].

It is estimated that patients affected by FA have a 500- to 1000-fold risk for HNSCC development, compared to the general population [4]. The risk for solid tumors in FA such as HNSCCs rises in early adulthood with a reported median between 20 and 32 years of age [5–7]. Chronic graft versus host disease arising in patients who have received HSCT is considered a major risk factor for HNSCC and is further complicated by long-term immunosuppression [6]. Primarily, HNSCC develops in the oral cavity, most commonly the tongue and gingival areas [5, 8]. The diagnosis is typically made late with locoregional lymph node positivity, and HNSCC in FA patients is considered to be aggressive in nature. Additionally, an increased risk for multiple primary malignancies has also been reported [4, 5].

As our case demonstrates, standard treatment modalities for HNSCC are not a straightforward choice for patients with FA. Surgery is the primary therapeutic approach. Large reconstructions, free flaps, and lymph node evacuations are tolerated with comparable morbidity to non-FA patients, whereas chemotherapy (CTx) and radiation therapy (XRT) are complicated due to their high toxicity, driven by the defective DNA repair in FA [9]. FA patients typically develop complications such as severe mucositis, dysphagia, and hematological abnormalities despite experimentally attenuated XRT dosing [7], but small patient cohorts have also reported positive treatment tolerance [10]. Adjuvant therapies must therefore be carefully evaluated in order to avoid severe systemic complications. Therefore, early identification and aggressive surgical management are of the utmost importance in HNSCC treatment in FA, although adjuvant therapies are not categorically contraindicated owing to the poor prognosis of HNSCC in this cohort.

Some reports have shown increased prevalence of oral leukoplakia on FA patients who did not undergo a HSCT [11]. Nevertheless, the literature concerning the prevalence of erytroplakia, leukoplakia, or lichen planus lesions on FA patients is scanty at best, demonstrating an urgent need for further studies on early detection of oral premalignant lesions in these patients [12].

Human papillomavirus (HPV) is a known risk factor of HNSCC. In FA patients, the situation is less clear. Higher HPV prevalence with a broad spectrum of HPV types has been reported for FA patients, and experimental evidence further points toward a potential association between HPV and HNSCC in FA. However, the etiologic connection between HPV and HNSCC in FA remains to be determined [8, 13]. Regardless, HPV vaccination should be recommended for all FA patients.

Recently, Valleuer and coworkers reported the advantages of oral brush biopsy-based cytology to identify oral SCC or its precursor lesions at an early stage. This landmark study included 1233 brush biopsies from an unprecedentedly large cohort of 713 FA patients. They concluded that careful inspection of the oral cavity followed by brush biopsy of suspected lesions detects 63% of SCCs and its precursors at an early stage and negative cytology, especially when combined with DNA ploidy analysis, resulted in a benign course with a high specificity [14]. The benefits of brush biopsies in FA include low cost and minimal invasiveness in addition to potentially excellent sensitivity and specificity. However, the study employed the laboratory and pathologists of an expert cytopathology department, also with rigorously controlled ploidy analysis methodology. Therefore, should other hospitals consider routine oral screening of FA patients, employing adequately experienced cytopathologists and establishing well-controlled laboratory methods are also warranted.

Lastly, owing to the complexity of SCC treatment and the aggressive nature of the disease, we suggest that from early adulthood, patients with Fanconi anemia undergo a biannual screening of the oral cavity. We consider regular oral examinations, optimally performed by a specialist in oral diagnosis in an office properly equipped for oral examination, the only effective method of facilitating early detection of oral premalignancy and carcinoma. All the risk factors for HNSCC should also be addressed, a personalized plan for oral hygiene instructed with motivation for tobacco and alcohol abstinence. HPV vaccination should be recommended. Should a patient present with a leukoplakia or other premalignant mucosal lesions, follow-up examinations should be performed every 6-8 weeks, as stated by Kutler et al. [7] in order to increase survival and minimize morbidity caused by HNSCC for patients with this rare disease [10, 13].

## Data Availability

No data were used to support this study.
